# Sensitive Detection of SARS-CoV-2–Specific Antibodies in Dried Blood Spot Samples

**DOI:** 10.3201/eid2612.203309

**Published:** 2020-12

**Authors:** Gabriella L. Morley, Stephen Taylor, Sian Jossi, Marisol Perez-Toledo, Sian E. Faustini, Edith Marcial-Juarez, Adrian M. Shields, Margaret Goodall, Joel D. Allen, Yasunori Watanabe, Maddy L. Newby, Max Crispin, Mark T. Drayson, Adam F. Cunningham, Alex G. Richter, Matthew K. O’Shea

**Affiliations:** University of Birmingham, Birmingham, UK (G.L. Morley, S. Taylor, S. Jossi, M. Perez-Toledo, S.E. Faustini, E. Marcial-Juarez, A.M. Shields, M. Goodall, M.T. Drayson, A.F. Cunningham, A.G. Richter, M.K. O’Shea);; The Saving Lives Charity, Birmingham (S. Taylor);; University Hospitals Birmingham NHS Foundation Trust, Birmingham (S. Taylor, A.M. Shields, A.G. Richter, M.K. O’Shea);; University of Southampton, Southampton, UK (J.D. Allen, Y. Watanabe, M.L. Newby, M. Crispin);; University of Oxford, Oxford, UK (Y. Watanabe)

**Keywords:** COVID-19, coronavirus disease, SARS-CoV-2, severe acute respiratory syndrome coronavirus 2, viruses, respiratory infections, zoonoses, antibody, dried blood spot, DBS

## Abstract

Dried blood spot (DBS) samples can be used for the detection of severe acute respiratory syndrome coronavirus 2 spike antibodies. DBS sampling is comparable to matched serum samples with a relative 98.1% sensitivity and 100% specificity. Thus, DBS sampling offers an alternative for population-wide serologic testing in the coronavirus pandemic.

A confirmed diagnosis of acute coronavirus disease (COVID-19) depends on the detection of RNA from the causative pathogen, severe acute respiratory syndrome coronavirus 2 (SARS-CoV-2). In contrast, although serologic testing is less useful for diagnosing the acute stages of infection, it can aid in diagnosing atypical manifestations of SARS-CoV-2 infection (M. Perez-Toledo et al., unpub. data, https://doi.org/10.1101/2020.06.05.20123117) and in determining prior virus exposure at a population level (*1*), knowledge which could substantially influence public health and social policies (*2*,*3*).

Currently, antibody testing for SARS-CoV-2 uses serum or plasma collected by venipuncture. The use of such sampling in large-scale seroepidemiologic studies is limited by logistic challenges, resources, and costs, as well as the risk for SARS-CoV-2 exposure from direct patient contact. In contrast, dried blood spot (DBS) sampling is simple, inexpensive, and can be self-collected and then sent by postal services to laboratories for processing (*4*). It is a well-established method for detecting antibodies against various infections (*5*,*6*), and antibodies collected by DBS are stable for prolonged periods (*7*). Moreover, DBS sampling provides a solution to widening access to serologic platforms in low- and middle-income countries. Nevertheless, the potential role of DBS sampling in studying SARS-CoV-2 seroprevalence has not been fully explored, and knowledge regarding the recovery of antibody from the DBS is limited. We describe the validation of DBS samples against matched serum in a highly sensitive and specific SARS-CoV-2 ELISA.

## The Study

We collected 87 samples from 80 volunteers at the University Hospitals Birmingham NHS Foundation Trust (under approved protocol for blood donations use in clinical assays, UK Research Ethics Committee reference no. 2002/201 and Clinical Immunology Service Reference no. ERN_16-178) during May 18–June 3, 2020. Three matched samples were from SARS-CoV-2 serum antibody–negative volunteers. The remaining samples were from SARS-CoV-2 serum antibody–unknown volunteers; 5 volunteers provided duplicate and 1 volunteer provided triplicate matched samples (Appendix Figure). To refine negative thresholds, we included 17 pre–August 2019 DBS-only samples (UK Research Ethics Committee reference no. 2002/20, Integrated Research Application System reference no. 132132, University Hospitals Birmingham project reference no. RRK4136). Volunteers were healthy at the time of sampling. Thirty-one matched samples (31/87 [35.6%]) were from PCR-positive volunteers, on average, 54 days (SD + 17 days) from reported symptom onset and 45 days (SD + 15 days) from PCR testing. All participants were anonymized, and SARS-CoV-2 PCR status was recorded as positive or unknown.

For DBS collection, we collected capillary blood samples onto forensic-grade 226 DBS cards (Ahlstrom Munksjo, https://www.ahlstrom-munksjo.com) by using finger-prick lancets (*4*,*8*). We stored DBS cards at room temperature in individual sample bags with desiccant. Concomitantly, we collected venous blood from volunteers and separated serum by using centrifugation at 9,700 × *g* for 5 min at room temperature. Laboratory analysis was blinded to PCR status, and we reported SARS-CoV-2–specific antibody results as positive, negative, or equivocal.

To elute antibody from DBS cards, we isolated individual preperforated DBS spots by using a sterile pipette tip and placed them into a universal tube at a ratio of 1 spot to 250 µL 0.05% phosphate-buffered saline (PBS)–Tween 20 (PBS-T) (PBS, Oxoid; Tween-20; Sigma-Aldrich, https://www.sigmaaldrich.com). We briefly vortexed and incubated tubes overnight at room temperature. We then harvested DBS eluate into a microtube and centrifuged it at 10,600 × *g* for 10 min at room temperature. We stored eluate at 4°C for <14 days in accordance with standard protocols (*4*). We quantified total IgG, IgA, and IgM concentrations in matched serum and DBS eluate, plus pre–August 2019 DBS samples, with nephelometry by using the automated COBAS 6000 (Roche, https://www.roche.com).

We performed a highly sensitive and specific in-house ELISA (now under peer review) to measure IgG, IgA and IgM against soluble, stabilized, trimeric SARS-CoV-2 spike (S) glycoprotein (*9*,*10*), as previously described (S.E. Faustini et al., unpub. data, https://doi.org/10.1101/2020.06.16.20133025). In brief, we coated Nunc 96-well plates (ThermoFisher, https://www.thermofisher.com) with 50 µL of 2 µg/mL S glycoprotein (M. Perez-Toledo et al.; S.E. Faustini et al.). We blocked plates and diluted samples with 2% BSA 0.1% PBS-T (PBS, Oxoid; Tween-20 and BSA, Sigma-Aldrich) at starting dilutions of 1:3 DBS eluate and 1:15 serum, with 3-fold serial dilutions; or single dilutions of 1:10 DBS eluate and 1:100 serum. We diluted mouse monoclonal anti–human horseradish peroxidase conjugated antibodies (anti–IgG R-10 1:8,000, anti–IgA MG4.156 1:4,000, and anti–IgM AF6 1:2,000; Abingdon Health, https://www.abingdonhealth.com) in 0.1% PBS-T. We developed plates with TMB Core (Bio-Rad, https://www.bio-rad.com) and stopped them after 5 min with 0.2M H_2_SO_4_ (Sigma-Aldrich). We recorded optical densities at 450 nm (OD_450_) by using the Dynex Revelation (Dynex Technologies, https://www.dynextechnologies.com). We reported results as SARS-CoV-2 S antibody positive, negative, or equivocal. The cutoff for negativity was less than the highest negative control (DBS 0.399 OD_450_ and serum 0.449 OD_450_), and for positivity, the mean of the negative controls +3 SD (DBS 0.444 OD_450_ and serum 0.62 OD_450_); a result between this range was considered equivocal.

We performed statistical analyses by using Prism 8 (GraphPad, https://www.graphpad.com) and assessed correlations between continuous data by using Spearman’s rank test (p<0.05 was considered statistically significant). We assessed DBS sample ELISA performance, relative to the serum assay, by calculating the comparative sensitivity, specificity, and positive and negative predictive values, with 95 % CIs. We assessed the agreement between DBS and serum ELISA results by determining the Cohen κ coefficient and Bland-Altman mean-difference.

**Figure 1:**
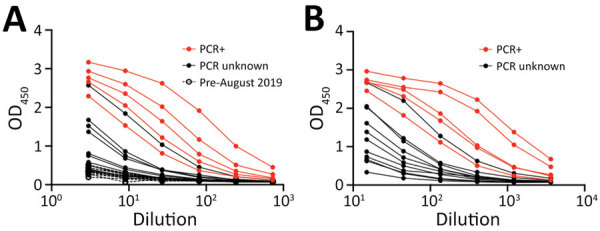
Elution of SARS-CoV-2 anti-spike glycoprotein antibodies from DBS samples, showing 3-fold DBS eluate (A) (initial 1:3 dilution) and serum (B) (initial 1:15 dilution) titrations. Dashed line indicates pre–August 2019 DBS samples (n = 11). Red circles indicate PCR-positive samples (n = 5). Black circles indicate PCR-unknown samples (n = 11), from matched contemporaneous samples. All samples were selected at random for inclusion. DBS, dried blood spot; OD_450_, optical density at 450 nm; SARS-CoV-2, severe acute respiratory syndrome coronavirus 2.

We performed quantification of total immunoglobulin concentrations in serum and DBS eluate. We observed 7- to 11-fold reduction in mean immunoglobulin concentration (IgG, IgA, and IgM) in DBS eluate compared with matched serum (Table 1). Matched serum and DBS titration curves showed the detection of SARS-CoV-2 S glycoprotein antibodies in both serum and DBS eluate with the limits of detection and the optimal detection dilution indicated (1:10 for DBS eluate and 1:100 for serum). PCR-positive matched samples showed higher responses, whereas pre–August 2019 DBS samples were negative across all dilutions (Figure 1).

**Table 1:** Mean concentrations of SARS-CoV-2 IgG, IgA, and IgM measured in matched DBS eluate and serum samples

Sample type	Mean immunoglobulin concentration, g/L*		
	IgG (range)	IgA (range)	IgM (range)
DBS	1.08 (0.17–2)	0.25 (0.1–0.6)	0.13 (0.1–0.3)
Serum	11.77 (8.18–18.59)	2.55 (1.5–5.2)	0.99 (0.3–1.5)

*DBS, dried blood spot; SARS-CoV-2, severe acute respiratory syndrome coronavirus 2. †Includes 10 matched DBS and serum and 5 pre–August 2019 DBS.

**Figure 2:**
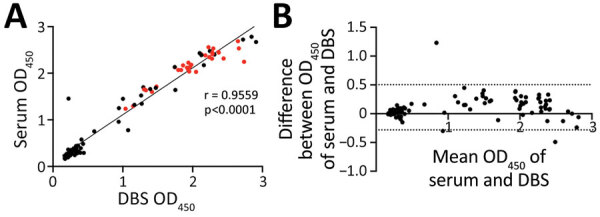
Effectiveness of DBS sampling for SARS-CoV-2 anti-spike glycoprotein detection. A) Correlation between matched DBS eluate (1:10) and serum (1:100) OD_450_ ELISA results (n = 87). Red circles indicate PCR-positive samples (n = 31). Black circles indicate PCR-unknown samples (n = 56). B) Bland-Altman mean-difference comparison of DBS eluate (1:10) and serum (1:100) OD_450_ ELISA results (dashed lines indicate 95% limits of agreement [−0.281 to 0.504]). DBS, dried blood spot; OD_450_, optical density at 450 nm; SARS-CoV-2, severe acute respiratory syndrome coronavirus 2.

We measured OD_450_ detected by ELISA for matched DBS eluate (diluted 1:10) and serum (diluted 1:100). We observed a significant correlation between matched serum and DBS samples (r = 0.96 [95% CI 0.93–0.97]; p<0.0001) (Figure 2, panel A) and minimal differences in results observed by sample type (Bland-Altman bias 0.11 + 0.20) (Figure 2, panel B). Discordance occurred between only 1 matched sample (κ = 0.975). Relative to serum samples, DBS samples achieved 98.11% sensitivity and 100% specificity for detecting S glycoprotein antibodies (Table 2); 100% of the PCR-positive samples (n = 31) were also antibody-positive in DBS eluate.

**Table 2:** 4x4 table of DBS eluate SARS-CoV-2 ELISA sensitivity and specificity, relative to serum samples*

Sample type		Serum	
		+	–
DBS	+	52	0
	–	1	31
Sensitivity, % (95% CI)		98.11 (89.93–99.95)	
Specificity, % (95% CI)		100 (88.78–100.00)	
PPV, %		100	
NPV, % (95% CI)		96.88 (81.65–99.54)	
Cohen’s kappa coefficient (95% CI)		0.975 (0.925–1.00)	

*Includes 87 matched DBS and serum samples tested for the detection of SARS-CoV-2 anti-spike glycoprotein; positive or negative matched samples (n = 84) were included, and equivocal results (n = 3) were excluded from the sensitivity and specificity analysis. DBS, dried blood spot; NPV, negative predictive value; PPV, positive predictive value; SARS-CoV-2, severe acute respiratory syndrome coronavirus 2.

## Conclusions

We show that DBS samples can be used for the detection of SARS-CoV-2–specific antibodies with results comparable to serum samples, supporting the findings of recent preliminary studies (*11*,*12*). Although individual laboratories should optimize DBS-derived antibody detection, considering dilution-factor and cutoff thresholds for their relevant downstream assay, these results demonstrate that DBS sampling could complement venipuncture for serologic assessments, such as seroprevalence studies, during the COVID-19 pandemic.

A current limitation of antibody assays is the necessity for venipuncture by skilled phlebotomists; DBS sampling overcomes this limitation and introduces the opportunity for wider population-level testing and improved surveillance in groups at heightened risk for infection. For example, DBS could be delivered using postal services (*4*) to patients with chronic conditions, the immunocompromised, and the elderly, all of which are groups disproportionately affected by COVID-19 (*13*). Furthermore, the DBS method is simple and inexpensive (*4*), which could enhance sampling in low- and middle-income countries, among groups where venipuncture is culturally unacceptable or in a geographically dispersed population.

Appendix Additional information about sensitive detection of SARS-CoV-2–specific antibodies in dried blood spot samples.
