# Supplementary material for: Sensitive Detection of SARS-CoV-2–Specific Antibodies in Dried Blood Spot Samples
Source: Emerg Infect Dis. 2020 Dec;26(12):2970–3. doi: 10.3201/eid2612.203309 (PMC7706975; doi:10.3201/eid2612.203309)
Supplement: Appendix — Additional information about sensitive detection of SARS-CoV-2–specific antibodies in dried blood spot samples. [file 20-3309-Techapp-s1.pdf]

Article DOI: https://doi.org/10.3201/eid2612.203309

Sensitive Detection of SARS-CoV-2–Specific Antibodies in Dried Blood Spot Samples

Appendix

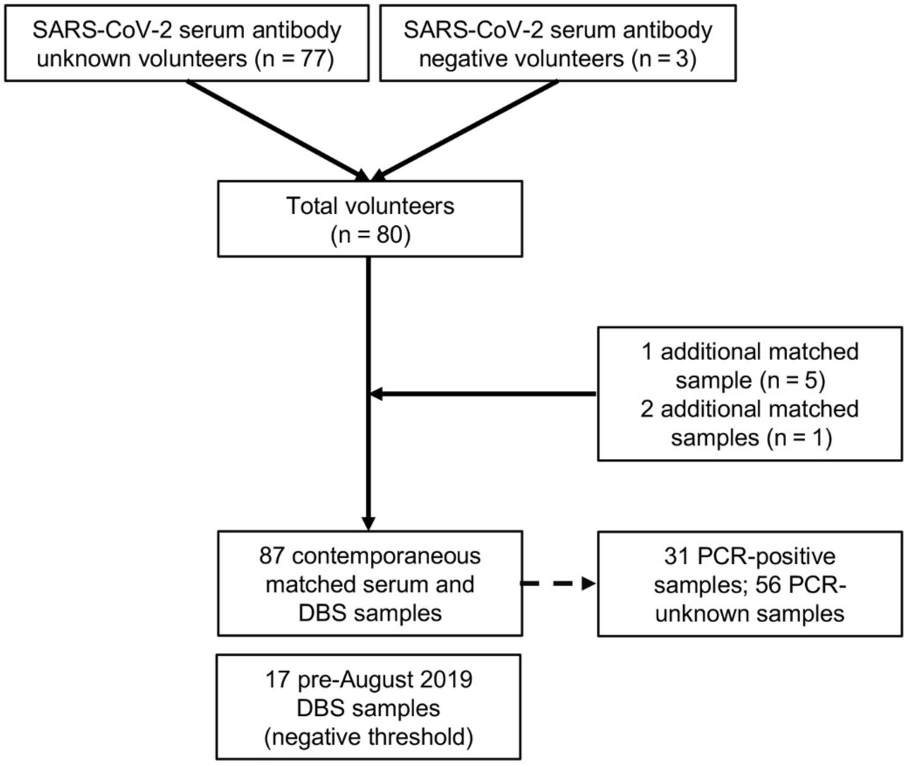

Appendix Figure. Study participant inclusion and sample selection. Eighty-seven matched contemporaneous samples were obtained from 80 healthy volunteers. Five volunteers gave one additional matched sample and one volunteer gave two additional matched samples. All 87 matched samples were included in the sensitivity, specificity, correlation and Bland-Altman analyses. For sensitivity and specificity calculations, three equivocal samples were excluded from the final analysis. Seventeen pre–August 2019 DBS samples were unmatched and used to refine negative thresholds only.
